# PubChemSR: A search and retrieval tool for PubChem

**DOI:** 10.1186/1752-153X-2-11

**Published:** 2008-05-15

**Authors:** Junguk Hur, David J Wild

**Affiliations:** 1 Bioinformatics Program, University of Michigan, Ann Arbor, MI 48109, USA; 2 Indiana University School of Informatics, 901 E. 10^th^ Street, Bloomington, IN 47406, USA

## Abstract

**Background:**

Recent years have seen an explosion in the amount of publicly available chemical and related biological information. A significant step has been the emergence of PubChem, which contains property information for millions of chemical structures, and acts as a repository of compounds and bioassay screening data for the NIH Roadmap. There is a strong need for tools designed for scientists that permit easy download and use of these data. We present one such tool, PubChemSR.

**Implementation:**

PubChemSR (Search and Retrieve) is a freely available desktop application written for Windows using Microsoft *.NET *that is designed to assist scientists in search, retrieval and organization of chemical and biological data from the PubChem database. It employs SOAP web services made available by NCBI for extraction of information from PubChem.

**Results and Discussion:**

The program supports a wide range of searching techniques, including queries based on assay or compound keywords and chemical substructures. Results can be examined individually or downloaded and exported in batch for use in other programs such as Microsoft Excel. We believe that PubChemSR makes it straightforward for researchers to utilize the chemical, biological and screening data available in PubChem. We present several examples of how it can be used.

## Background

Recent years have seen an explosion in the amount of chemical and related biological information in freely-accessible databases [1,2] The most widely known of these is PubChem [3], a repository of over 40 million chemical substances (at the time of writing) with associated property, literature reference and biological activity information. In addition to being a resource of information about compounds, this database is the primary repository for High Throughput Screening results generated by the Molecular Libraries Screening Centers Network (MLSCN) [4], part of the NIH Roadmap.

While PubChem has a straightforward web-based user interface for searching, it is quite limited in its facilities for download and processing of search results. For example, one can download data for a particular PubChem entry in XML [5] and a few other formats, but it is not possible to download aggregate search results in a manner that is straightforward for a non-computational scientist. Yet the greatest utility of this information is clearly in aggregate: with structural information for compounds tested in a particular bioassay, one can create a QSAR model; by comparing compounds active in one assay with those active in a second, one can make judgments about selectivity; by downloading properties for compounds similar to a query one can investigate the behavior of a series of compounds rather than individual compounds. There is thus a need for tools to be developed that allow easy search, access and download of information in PubChem, and in particular which allow one to move information *en bloc *to one's own computer for further processing. The development of PubChemSR was thus driven by the desire to have at hand such features as:

• Easy search and retrieval of detailed compound, substance and bioassay information, including substructure and similarity searching

• Interactive refinement of searches

• Facility to export information to simple text or Microsoft Excel files and to specifically include or exclude individual data fields

• The ability to easily retrieve compounds that are active or inactive (or both) in particular bioassays

## Implementation

PubChemSR (Search and Retrieve) is written in Microsoft .NET Visual Basic 2005 [6] and retrieves information from the PubChem database using the NCBI Entrez [7] web service version 1.5a via a SOAP interface [8]. It is compatible with Windows XP and the newer Windows Vista. We chose .NET [9] as it enables the maximum flexibility in design of user interface, and makes use of the SOAP protocol straightforward. The major limitation of this approach is that the program can only be used in a Windows environment.

The Microsoft .NET Framework is a software component which provides a plethora of pre-coded solutions to common software development requirements, and manages the execution of applications written for the framework. The deployment size of an application is small since the application can be executed in the runtime environment with .NET framework installed on a user's side.

SOAP (Simple Object Access Protocol or lately also know as Service Oriented Architecture Protocol) is a protocol allowing XML (Extensible Markup Language) based communication over computer networks using the World Wide Web's Hypertext Transfer Protocol (HTTP). One advantage of using SOAP is that it allows easier communication through firewalls and proxies since SOAP runs through HTTP requests that ensure unblocked communication with other programs anywhere. SOAP is one of the languages that enable the deployment of web services for remote access and execution of code. Web services have proven useful in both bioinformatics, and more recently, in cheminformatics [10] for the flexible interaction of distributed data and computation components.

NCBI provides a collection of web services that allow programmable access and query to the Entrez data. These Entrez Programming Utilities, or eUtils, include EInfo, ESearch, EPost, ESummary, EFetch, ELink, EGQuery, ESpell and they are all wrapped into SOAP interface for easier communication. This is the primary mechanism used by PubChemSR for data retrieval. For structure search and BioAssay data retrieval that is not supported through the NCBI SOAP interface, PubChemSR performs such tasks in the background by directly accessing the NCBI's web server.

The JME (Java Molecular Editor) [11] written by Peter Ertl of Novartis is used to draw structure queries and to convert them into SMILES strings. PubChemSR allows users to interact with the JME applet at the PubChemSR web page [12] or the standalone version that comes with the PubChemSR distribution package. The latter requires the JAVA runtime environment to be available on a user's machine [13].

## Results and discussion

### Search Modes

PubChemSR employs a GUI (Graphical User Interface) with reasonably self-explanatory sections and buttons. It currently supports the three different search modes: simple text search mode (in the main window), structure search mode (in the main window), and batch search mode (through the Tools menu). The simple text search mode and structure search mode provide the same search functionality as the NCBI's Entrez or PubChem basic structure search, while the batch search mode extends the batch Entrez in ways enabling users to run a list of queries and merge the results into a single file.

### URL Analyzer

URL Analyzer can retrieve search results and display them in the search result view panel after users perform searches in their web-browser. The full URL of the results web-page can be copied into the clipboard using 'Copy' or 'Ctrl+C'. The user can then paste the URL into the URL analyzer by clicking the *Get *button or by pasting into the box. The *Anal *button will check the URL and retrieve the search results into the preview panel. This feature becomes extremely useful when a search can not be completed within a specified time (default is 120 seconds) or is not supported in PubChemSR. Such examples include structure searches for similar/substructure compounds or advanced structure searches supporting additional filters like chemical property or BioActivity.

### Bulk Download

Bulk download enables users to download information on compounds *en masse *and only export the desired data fields for each compound. Needed are a list of UIDs (Unique Identifiers: CID for compounds, SID for substances, and AID for BioAssay), which can be obtained through the simple text search or be uploaded from a file. The buttons in the 'Retrieve' panel will either directly save the data into a text file or display them first in a separate window giving further options to export the data into Microsoft Excel or HTML file.

### Other features

Several other available features are offered by the program including *term correction for misspelled queries *– misspelled queries can be automatically corrected via NCBI E-spell web-service; *selectable data field *– for bulk download, the results can be filtered to only include fields of interest to the user; *preview with picture *– the search result view panel provides a summary of the results ten compounds at a time with preview of structure and selected data fields; and *BioAssay retriever *– retrieves the actual bioassay activity data and exports them along with selected compounds/substance data fields to Microsoft Excel or text files.

### Examples of Use

There are many ways that PubChemSR can be used to simplify the process of obtaining information from PubChem. Below are listed a few examples of how it can be employed for common tasks.

#### Comparing chemical properties of related compounds

**Figure 1:**
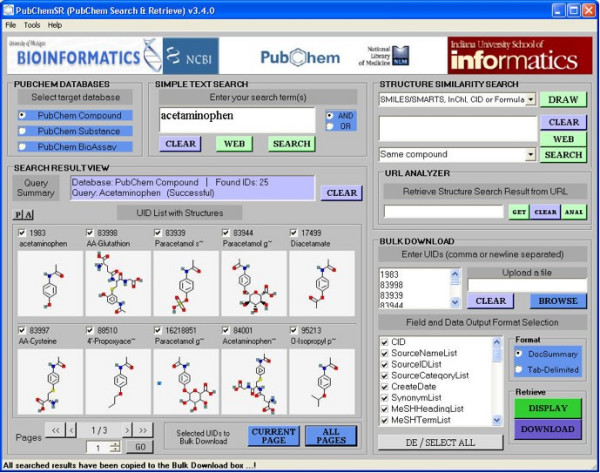
**Main window of PubChemSR**. Shown is a simple search result of 'acetaminophen' at the PubChem compound database. PubChemSR has retrieved 25 records by 'acetaminophen' at the PubChem compound database. The bottom left panel shows the structure of the retrieved compounds (10 per page). Clicking 'Current Page' or 'All Pages' will copy the selected (checked) UIDs to the Bulk-download section for further downloading of the full or selected data field.

**Figure 2:**
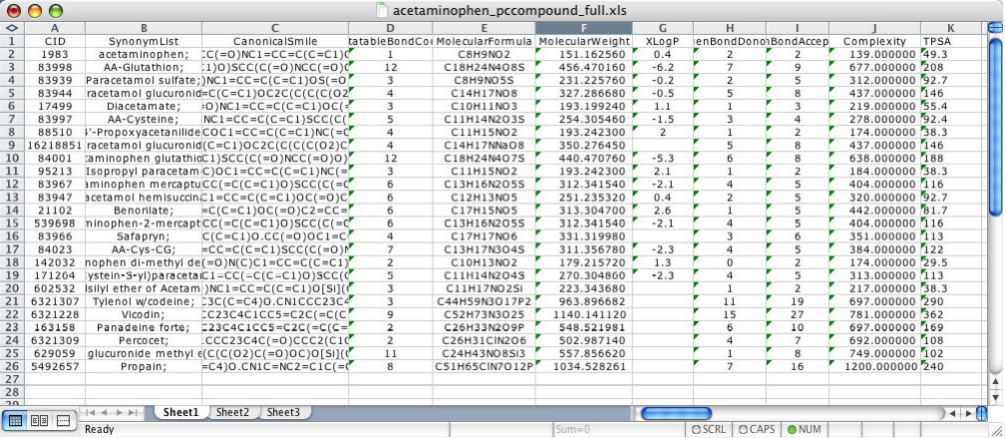
**Property data exported into Microsoft Excel**. Selected property-related fields of the 25 'acetaminophen' related compounds were exported into an Excel file. The filtering, sorting and graphing features of Excel can then be used to examine this data.

It is often useful to compare the properties of compounds in a particular structural class. This is very easy to do using the refinement and Excel export functions. Figures 1 and 2 show respectively a search for 'acetaminophen' using PubChemSR, and an Excel spreadsheet created by exporting selected property-related fields from the program. This kind of comparison may also be done with a substructure or similarity search instead of a simple text search.

#### Browsing bioassays related to kinases, and downloading active compounds in specific assays

**Figure 3:**
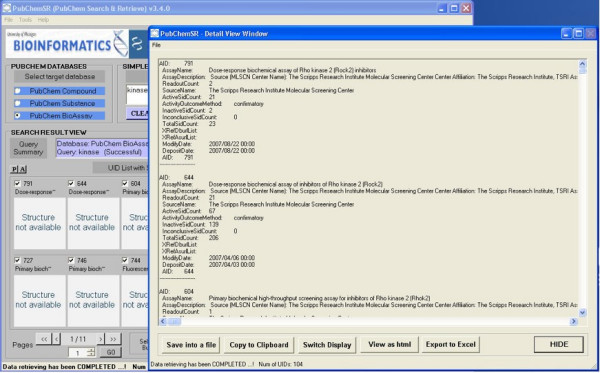
Browsing the results after a search of the bioassay database for kinase-related assays.

**Figure 4:**
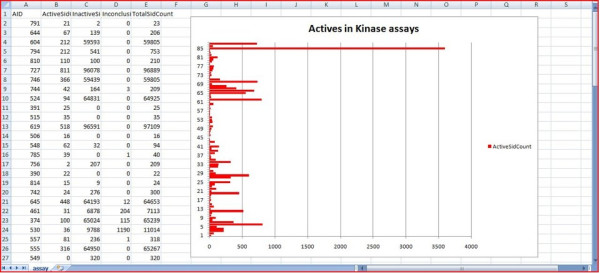
**An analysis of kinase-related assays in Excel**. Here a graph is used to compare the numbers of active compounds in each of the assays.

**Figure 5:**
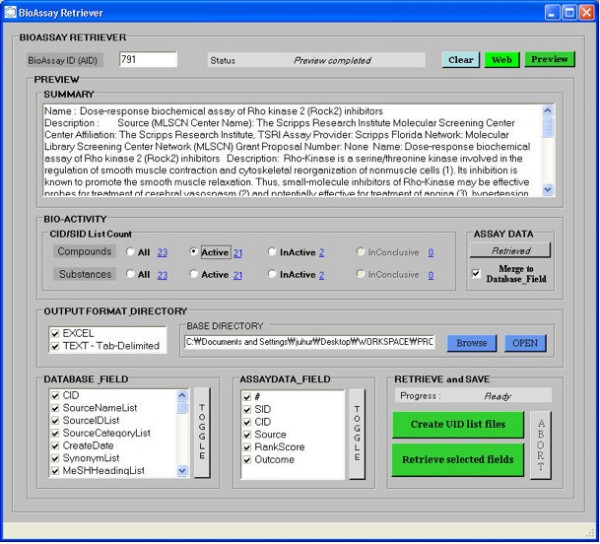
Retrieving active and inactive compounds for a particular assay using the bioassay retriever in PubChemSR.

Using a text search on the PubChem BioAssay database, one can find all of the assay descriptions that contain particular keywords such as "*Kinase*". One can then export all of these descriptions to Excel or a text file, or browse them from within the program (as shown in Figure 3) In particular, one can download statistics of assays (counts of active and inactive structures and so on) and use Excel to analyze these (see Figure 4). Upon finding assays of interest, one can retrieve all of the compounds (and related information) that have been flagged as showing activity in that assay by supplying the assay ID to the bioassay retriever as shown in Figure 5. These compounds can then be exported just as with a regular compound search.

#### Creating a SMILES and activity file for SAR study of an assay

*SMILES *is a linear text string representation of the 2D chemical structure of a compound. A SMILES file usually contains the SMILES string and name for a compound. When a third column is added that contains biological activity values for a compound, it is a useful format for input into a variety of cheminformatics techniques that can automatically determine structure-activity relationships (SAR) in compounds. Using the BioAssay Retriever, one can download just the SMILES, name, and biological assay results for compounds and then create a simple tab-delimited file that can be loaded into cheminformatics tools.

## Conclusion

We believe PubChemSR is an extremely useful and straightforward tool that bridges a gap between the needs of bench scientists and the rich information resource of PubChem. We have shown how it can be used to export and explore compound, property and bioassay information in the database. PubChemSR is not intended to replace the web-based PubChem interface, and there are certain features which are only available in the web-based PubChem interface such as structure clustering or structure-activity analysis in detailed BioAssay summary pages. PubChemSR has been designed to aid users, especially non-computationally experienced, to search, retrieve, export, and manipulate the PubChem data in more efficient and convenient ways.

## Availability and requirements

Project name: PubChemSR

Project home page: ; 

Operating system: Windows XP or Vista

Programming language: Microsoft Visual Basic .NET

Other requirements: Microsoft .Net 2.0

License: GNU General Public License version 3 .

Any restrictions on use by non-academics: The tool may not be used for commercial purposes

## Authors' contributions

The program was fully developed by JH initially under the supervision of DW. Both contributed to this paper.
